# Assessment of ventricular mechanical synchronization after left bundle branch pacing using 2‐D speckle tracking echocardiography

**DOI:** 10.1002/clc.23481

**Published:** 2020-10-21

**Authors:** Zhijun Sun, Beibing Di, Huikuan Gao, Dihui Lan, Hui Peng

**Affiliations:** ^1^ Department of Cardiology, Cardiovascular Center, Beijing Friendship Hospital Capital Medical University Beijing China

**Keywords:** left bundle branch pacing, right ventricle pacing, systolic synchronization, two‐dimensional strain echocardiography

## Abstract

**Background:**

The left bundle branch pacing (LBBP) makes the ventricular depolarization closer to the physiological state and shortens QRS duration. The purpose of this study is to explore the ventricular systolic mechanical synchronization after LBBP in comparison with traditional right ventricular pacing (RVP) using two‐dimensional strain echocardiography (2D‐STE).

**Methods:**

Thirty‐two patients who received LBBP (n = 16) or RVP (n = 16) from October 2018 to October 2019 and met the inclusion criteria were included in this retrospective study. Electrocardiogram (ECG) characteristics, pacing parameters, pacing sites, and safety events were assessed before and after implantation. Acquisition and analysis of ventricular systolic synchronization were implemented using 2D‐STE.

**Results:**

In RVP group, ECG showed left bundle branch block patterns. At LBBP, QRS morphology was in the form of right bundle branch block, and QRS durations were significantly shorter than that of the RVP QRS (109.38 ± 12.89 vs 149.38 \± 19.40 ms, *P* < .001). Both the maximum time differences (TD) and SDs of the 18‐segments systolic time to peak systolic strain were significantly shorter under LBBP than under RVP (TD, 66.62 ± 37.2 vs 148.62 ± 43.67 ms, *P* < .01; SD, 21.80 ± 12.13 vs 52.70 ± 17.72 ms, *P* < .01), indicating that LBBP could provide better left ventricular mechanical synchronization. Left and right ventricular pre‐ejection period difference was significantly longer in RVP group than in LBBP group (10.23 ± 3.07 vs 39.94 ± 14.81 ms, *P* < .05), indicating left and right ventricular contraction synchronization in LBBP group being better than in RVP group.

**Conclusion:**

LBBP is able to provide a physiologic ventricular activation pattern, which results in ventricular mechanical contraction synchronization.

## 1 INTRODUCTION

The traditional pacing site at the right ventricular apex or the right side of ventricular septum was demonstrated to have detrimental impact on clinical outcomes due to ventricular mechanical dyssynchrony secondary to electrical dyssynchrony.^1^, ^2^ Studies have demonstrated the feasibility and clinical benefits of permanent His‐bundle pacing (HBP).^3^ Former researchers have found that permanent HBP led to significant narrowing of QRS duration and improvement in the left ventricle (LV) function in patients with reduced LV ejection fraction (LVEF).^4^ But His bundle pacing still have several disadvantages, including difficulty of lead implantation, lower R‐wave amplitudes, high and unstable pacing threshold, especially in patients who have conduction block distal to the His bundle.^3^, ^5^ Upadhyay et al demonstrated that the site of block usually was located within the His or proximal left bundle.^6^

Thus, alternative pacing sites have been sought. After penetrating through the membranous atrioventricular septum, the conductive fibers of the left bundle branches spread beneath the endocardium of ventricular septum in a relatively large dimension,^7^ which offers an opportunity for pacing the left bundle branch (LBB) in an easier manner. Weijian Huang et al developed a technique for the left bundle branch pacing (LBBP) using a transseptal approach.^8^ LBBP has been reported to offer low pacing thresholds and large R waves, and has a lower theoretical risk for the development of distal conduction block due to that the distal conduction system is targeted.^9^ Keping Chen et al reported that LBBP had a lower pacing threshold and produced narrower electrocardiogram (ECG) QRS duration compared with the right ventricular pacing (RVP).^10^ The purpose of this study is to explore the electrical and mechanical synchrony of the ventricle using two‐dimensional strain echocardiography (2D‐STE) after LBBP in comparison with the traditional RVP.

## 2 METHODS

### 2.1 Study design

This study was performed in Beijing Friendship Hospital with patients who were diagnosed as II° atrioventricular block (AVB), III° AVB, or sick sinus syndrome, and indicated for pacing therapy according to 2013 ESC/EHRA Guidelines.

The inclusion criteria were as follows: (a) age > 18 years; (b) II° AVB, III° AVB or I° AVB sick sinus syndrome with ventricular pacing ratio > 70%; (c) LVEF >50%; (d) New York Heart Association (NYHA) score of I or II; and (e) being able to visit the hospital during follow‐up. The exclusion criteria were as follows: (a) moderate to severe valvular diseases; (b) congestive heart failure; (c) acute or old myocardial infarction; (d) history of cardiomyopathy; (e) poor condition of the acoustic window because of emphysema or other reasons; or (f) severe liver, lung or kidney dysfunction.

The protocol was approved by Beijing Friendship Hospital Institutional Review Board, and all patients had submitted written informed consent. From October 2018 to October 2019, 32 patients who received LBBP or RVP and met the criteria mentioned above were included.

### 2.2 Implantation procedures

Before the pacemaker implantation, the ventricular septal thickness was assessed by Echo. Twelve‐lead ECG and intracardiac electrograms were simultaneously displayed and recorded on a multichannel recorder.

**FIGURE 1:**
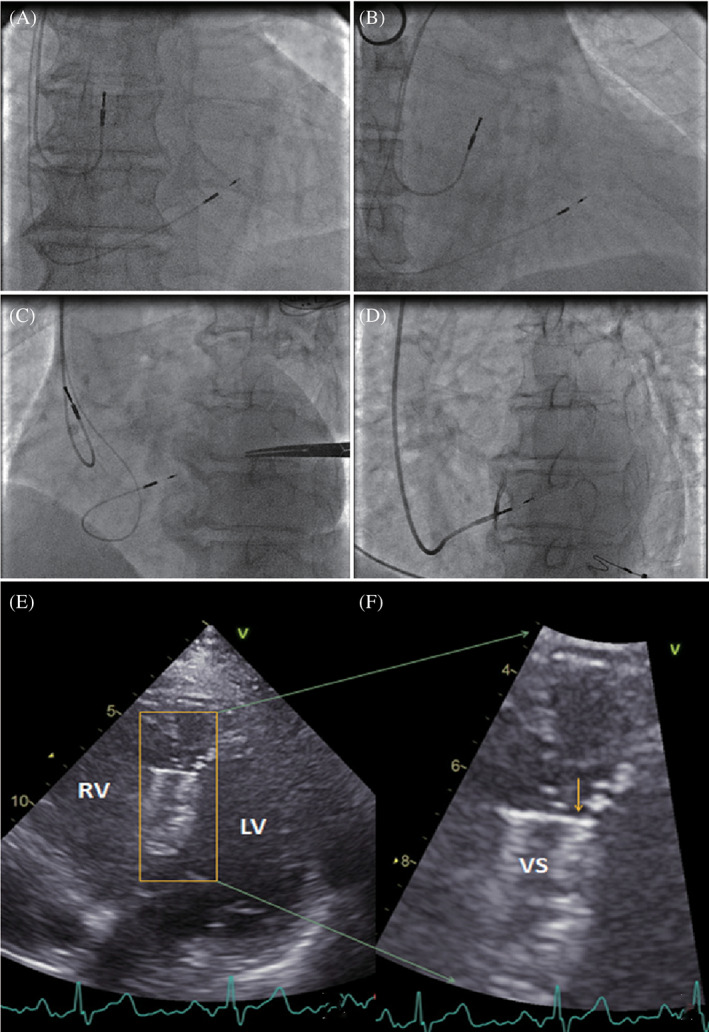
LBBP lead location confirmed by fluoroscopy and post‐implant 2D echocardiography. Fluoroscopic imaging when the pacing lead was placed in the left bundle branch area which was beneath the left side of the ventricular septum in orthotopic view, the right anterior oblique 30° view and the left anterior oblique 40° view, A‐C. Contrast injection through the sheath in the left anterior oblique 35° view, D. F, Enlarged picture of, E, to show the pacing lead tip under the left endocardium of the septum. The arrow indicates the location of the pacing lead tip with a high density. LBBP, left bundle branch pacing; LV, left ventrium; RV, right ventrium; VS, ventricular septum

LBBP: The delivery sheath (C315 HIS, Medtronic Inc., Minneapolis, MN) was inserted via the left subclavian vein or axillary vein and placed on the right side of the septum inferior to the septal leaflet of the tricuspid valves about 1 to 1.5 cm from HBP site toward right ventricular apex (RAO 30°). Then, the Select Secure pacing lead (Model 3830 69 cm, Medtronic Inc., Minneapolis, MN) was advanced through the sheath with its tip just beyond the distal part of the sheath for unipolar pacing and local activation potential recording. The sheath and the pacing lead touched the septum and pacing with an output of 5.0 V/0.4 ms was applied, which created ECG QRS morphology of “W” pattern with the notch closer to nadir in lead V1. The pacing lead was then screwed perpendicularly to LV septum (LAO 30°‐45°, Figure 1C). During the lead advancement procedure, the changes in the notch in V1 lead, the fulcrum sign, and impedance changes were observed, and sometimes the sheath angiography was used to determine LBBP lead depth into ventricular septum (Figure 1D). Once ECG QRS morphology during pacing resulted in a pattern of the right bundle branch block (RBBB) or a very narrow QRS complex (<120 ms), the lead had been at or near the left bundle branch and the lead advancement was stopped. Then the test with different output was used to confirm LBB capture. Evidences for direct LBB capture were as follows: (a) pacing morphology of RBBB pattern; (b) recording LBB potential; (c) stimulus‐peak R_V5_ or R_V6_ shortening abruptly with increasing output or remaining shortest and constant (<90 ms) at low and high outputs; (d) selective LBBP and non‐selective LBBP; or (e) recording retrograde His potential or anterograde LBB potential during pacing (not routine in clinical practice).^11^ The pacing parameters were measured to confirm stable capture threshold and consistent pacing impedance, and then the sheath was removed.

RVP: the pacing lead (models 5076, Medtronic Inc., Minneapolis, MN) was positioned in the right ventricular apex (n = 7, the right ventricular apical pacing [RVAP]) or the right side of ventricular septum (n = 9, right ventricular septal pacing [RVSP]). The lead positions in patients with LBBP and RVP were confirmed with the high‐quality fluoroscopic radiographs. All patients underwent the regular follow‐up postimplantation.

### 2.3 Programming

Conventional lead‐related parameters including stimulation threshold, impedance, and sensing amplitude were recorded right after implantation and 7 days later. Ventricular pacing rates of all patients were more than 70%. The atrioventricular delay was selected, which corrected bundle branch block and yielded narrower QRS duration. Lead‐related adverse events were not observed during follow‐up.

### 2.4 Echocardiographic parameters

Echocardiography was performed using VVIq ultrasound (GE Company, USA) with S‐5 transducers 7 days after surgery. Acquisition and analysis of synchronization using 2D‐STE were performed by senior sonographers. Standard apex four‐chamber view, apex three‐chamber view, and apex two‐chamber view were clearly exposed and recorded as well as ECG, and four consecutive cardiac cycles with constant heart rate were collected. The 18‐segment systolic times to peak 2‐D longitudinal systolic strain were recorded for every patient. Then the maximum time difference of systolic times to peak 2‐D strain among the 18 LV segments (2D‐TD_max_), and the SDs of systolic times to peak systolic strain (PSS) of the 18 LV segments were calculated. The 6‐segment time differences (TDs) to PSS of the apex four‐chamber view, apex three‐chamber view, and apex two‐chamber view were also calculated, respectively. Both the LV function and synchronization status using 2D‐STE were assessed using the offline software TOMTEC (Tom‐Tec Imaging Systems, Unterschleissheim, Germany). Septal‐to‐posterior wall motion delay (SPWMD) was also recorded (Supplementary Figure S1). Interventricular mechanical delay (IVMD) was used to reflect the synchrony between the left and right ventricles. IVMD was the time difference between the left and right ventricular pre‐ejection periods，which were the periods from the start of QRS wave to the start of aortic valve blood flow or pulmonary valve blood flow (Supplementary Figure S2).

### 2.5 Statistical analysis

Data were presented as mean ± SD for continuous variables. Quantitative data with normal distribution compared between two groups were evaluated using the Student *t* test. Quantitative data inconsistent with normal distribution compared between two groups were evaluated using the Wilcoxon rank sum test. Categorical data compared between two groups were evaluated using Fisher exact probabilities method. All *P* values were two‐tailed, and *P* values <.05 were considered to indicate statistical significance. All statistical analyses were performed using IBM SPSS Statistics 26.

## 3 RESULTS

### 3.1 Baseline characteristics

Thirty‐two patients with good acoustic windows were enrolled from October 2018 to October 2019. Sixteen patients were analyzed in each group. Among these patients (mean age = 71.4 ± 14.4 years in the LBBP group and 73.6 ± 8.9 years in the RVP group; *P* = .910), 27 were diagnosed with AVB, 6 with sick sinus syndrome, 3 with coronary diseases, 8 with hypertension, 11 with diabetes mellitus, and 2 with paroxysmal atrial fibrillation. No statistical differences were noted in baseline clinical characteristics between the two groups (Table 1).

**TABLE 1:** Patient baseline characteristics, and pacing and electrocardiogram parameters after LBBP and RVP

Groups	RVP (n = 16)	LBBP (n = 16)	*P* value
Demographics			
Age (years)	73.6 ± 8.9	71.4 ± 14.4	.910
Sex (Male/Female)	5/11	7/9	.716
Heart rate (beats/min)	55.3 ± 13.3	58.3 ± 14.4	.545
Comorbidities			
Coronary disease (yes/no)	2/14	1/15	1.0
Hypertension (yes/no)	4/12	4/12	1.0
Paroxysmal atrial fibrillation (yes/no)	0/16	2/14	.484
Diabetes mellitus (yes/no)	4/12	7/9	.458
Other diseases (yes/no)	2/14	1/15	1.0
Diagnosis			
SN dysfunction (yes/no)	3/13	3/13	1.0
AVB (yes/no)	14/2	13/3	1.0
LBBB (yes/no)	2/14	1/15	1.0
RBBB (yes/no)	3/13	2/13	1.0
Pacing parameters			
Sensing amplitude (mV)	12.37 ± 3.65	9.81 ± 4.08	.052
Pacing impedance (Ω)	678.38 ± 155.53	705.37 ± 133.51	.545
Pacing threshold (V)	0.78 ± 0.19	0.71 ± 0.19	.283
Pacing ECG parameters			
Baseline ECG QRS duration (ms)	107.50 ± 28.17	106.25 ± 25.00	.739
Paced ECG ORS duration (ms)	109.38 ± 12.89	149.38 ± 19.40	<.01
Pacing‐R wave peak of V5 lead (ms)	73.44 ± 7.00	97.19 ± 8.36	<.01
Pacing‐R wave peak of V6 lead (ms)	75.31 ± 7.85	102.81 ± 8.56	<.01

Abbreviations: AVB, atrium ventricular block; ECG, electrocardiogram; LBBB, left bundle branch block; LBBP, left bundle branch area pacing; RBBB, right bundle branch block; RVP, right ventricular pacing; SN dysfunction, sinus node dysfunction.

### 3.2 ECG characteristics, lead parameters, and location

**FIGURE 2:**
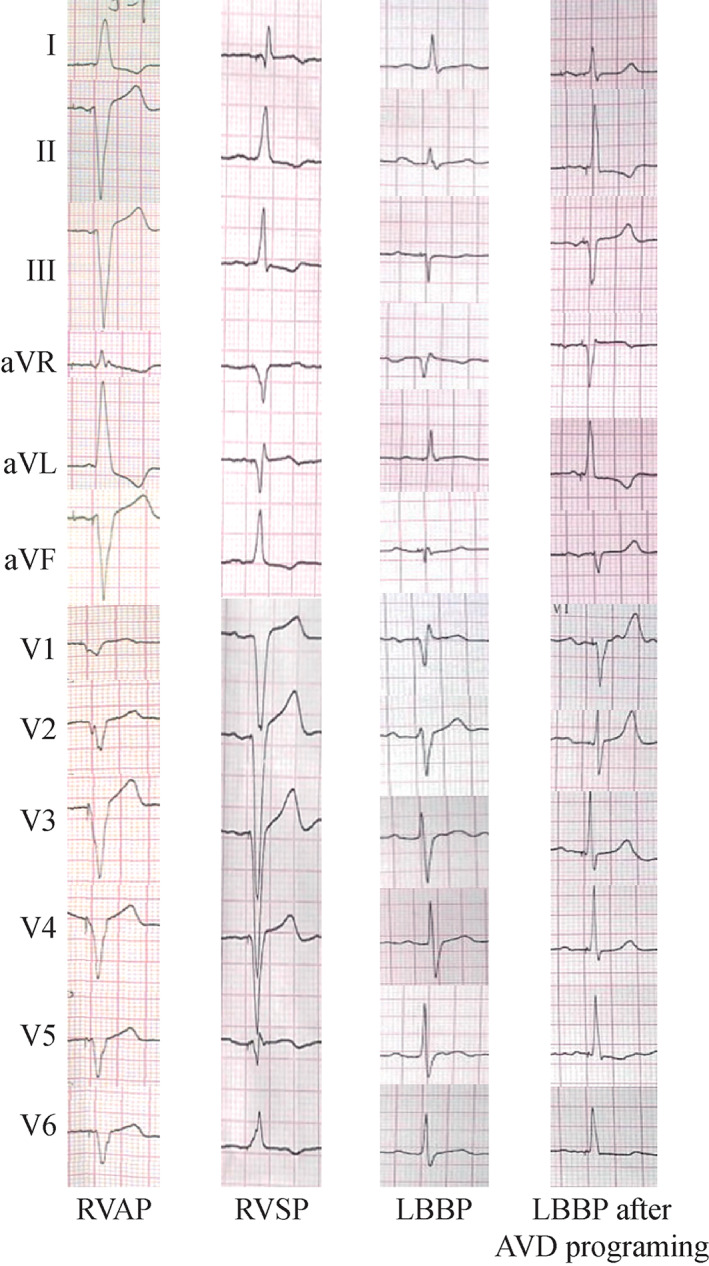
Twelve‐lead ECG after LBBP, RVAP, and RVSP. QRS duration of LBBP post‐implantation was much shorter than that of RVAP and RVSP. We can get narrower ORS waves by programing the AVD in order to fuse right and left ventricular depolarization after pacing. AVD, atrium ventricular delay; ECG, electrocardiogram; LBBP, left bundle branch pacing; RVAP, right ventricular apical pacing; RVSP, right ventricular septal pacing; RVP, right ventricle pacing

ECG was characterized with wide QRS complex and the pattern of LBBB)or delay during either RVAP or RVSP as shown in Figure 2. The LBB potentials were recorded in 8 of 16 patients who received LBBP. The interval from LBB potential to the beginning of ECG QRS was 21.62 ± 6.28 ms. The pacing spike to R wave peak of V5 and V6 leads were 73.44 ± 7.00 and 75.31 ± 7.85 ms, respectively, in the LBBP group. In all 16 patients receiving LBBP, there were equipotential lines between the pacing spike and the QRS wave, and the pacing spike to the beginning of ECG QRS was 28.48 ± 5.69 ms. In patients receiving RVAP or RVSP, ECG QRSs were immediately initiated by pacing without an equipotential line. ECG QRS duration was much shorter in LBBP group compared with that in RVP group (109.38 ± 12.89 vs 149.38 ± 19.40 ms, *P* < .01), but there was no significant difference in QRS duration between patients of LBBP group and RVP group at baseline.

There was no significant difference in sensing amplitude, pacing impedance, and capture threshold among LBBP and RVP (Table 1). Pacing thresholds and sensing amplitude measured at time of hospital discharge remained stable compared with that measured at implantation.

In patients with LBBP, fluoroscopy at implantation confirmed the location of the pacing lead in the left bundle branch area (Figure 1A‐D), and postimplantation echocardiography confirmed the location of the pacing lead on the left side of the basal septum (an example shown in Figure 1E‐F). Thus, LBBP in general is left septal subendocardial pacing. The echocardiography estimated distance from the lead tip to the right side of the septum in the LBBP group was 0.8 to 1.5 cm depending on the thickness of the basal ventricular septum.

### 3.3 LV synchronization status

**FIGURE 3:**
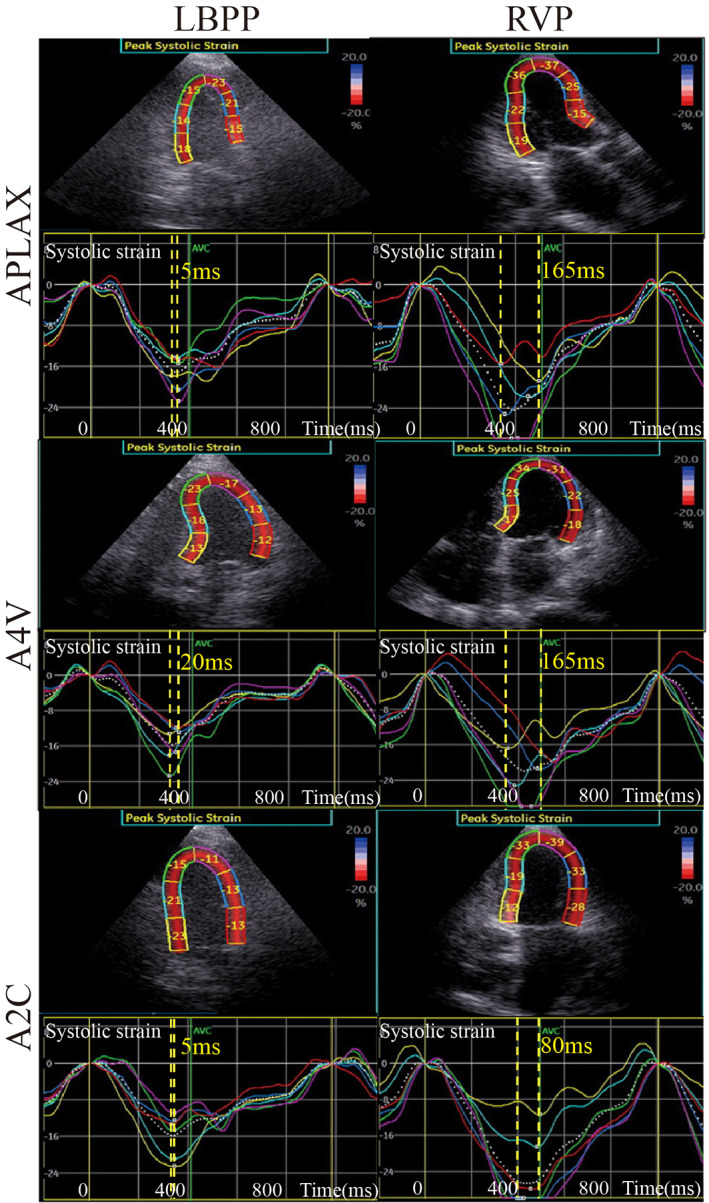
2‐D speckle‐tracking echocardiography was used to get the time‐systolic strain curve of the 18 segments. We calculate the maximum time difference of the 18‐segment systolic time to peak 2‐D strains (2‐D‐TD). Maximum 2‐D‐TD between ventricle segments in the RVP group was larger than those in the LBBP group. The TD to PSS of the six segments of the apex four‐chamber view, apex three‐chamber view, and apex two‐chamber view were larger in the RVP group than those in the LBBP group. LBBP, left bundle branch pacing; RVP, right ventricular pacing

The LV wall was divided into 18 segments, each of which showed individual systolic time to peak 2‐D longitudinal strain (Figure 3). Postoperatively, 18‐segment maximum time difference to peak 2‐D strain (2D‐TD_max_) was significantly shorter in the LBBP group than in the RVP group (66.62 ± 37.24 vs 148.62 ± 43.67 ms, *P* < .01); while at baseline, 2D‐TD_max_ did not differ between the two groups (51.69 ± 28.97 vs 50.13 ± 25.66 ms, *P* = .650). There was no statistical difference for 2D‐TD_max_ in the LBBP group when comparing with baseline (66.62 ± 37.24 vs 51.69 ± 28.97 ms, *P* > .05). After pacemaker implantation, the 2D‐TD_max_ was significantly longer compared with baseline in the RVP (148.62 ± 43.67 vs 50.13 ± 25.66 ms, *P* < .01). Similar results were obtained when comparing SD of 18‐segment systolic times to peak 2‐D strain between the two groups at baseline and after operation (before pacing 17.57 ± 10.04 vs 13.30 ± 8.14 ms, *P* = .187; after pacing 21.80 ± 12.13 vs 52.70 ± 17.72 ms, *P* < .01). There was no statistical difference for SD in the LBBP group when comparing with baseline (21.80 ± 12.13 vs 17.57 ± 10.04 ms, *P* > .05). After pacemaker implantation, the SD was significantly longer compared with baseline in the RVP (52.70 ± 17.72 vs 13.30 ± 8.14 ms, *P* < .01). (Table 2).

**TABLE 2:** Comparison of synchronization status and left ventricular function between the LBBP and RVP groups before and after pacemaker implantation

	Baseline		*P* value	After pacemaker implantation		*P* value
Groups	LBBP, n = 16	RVP, n = 16		LBBP n = 16	RVP n = 16	
EDD	5.07 ± 0.25	5.20 ± 0.25	.113	5.10 ± 0.25	5.24 ± 0.22	.097
LVEF (%)	68.69 ± 3.14	66.13 ± 4.50	.097	66.94 ± 1.69	65.19 ± 3.43	.136
Average of peak longitudinal 2D‐stain (−%)	21.36 ± 2.05	20.10 ± 4.26	.720	20.59 ± 2.44	18.90 ± 5.29	.474
SPWMD (ms)	36.00 ± 21.48	32.50 ± 18.58	.213	43.31 ± 23.27	93.56 ± 36.77	<.01
left and right ventricular pre‐ejection period difference (ms)	11.38 ± 7.79	12.88 ± 6.99	.484	10.23 ± 3.07	39.94 ± 14.81	<.01
18‐segment maximum time difference to peak 2‐D strain (ms)	51.69 ± 28.97	50.13 ± 25.66	.650	66.62 ± 37.24	148.62 ± 43.67	<.01
standard deviation of 18‐segment systolic times to peak 2‐D strain (ms)	17.57 ± 10.04	13.30 ± 8.14	.187	21.80 ± 12.13	52.70 ± 17.72	<.01
Anteroseptal segment vs posterior segment (ms)	23.81 ± 10.44	19.81 ± 9.98	.255	22.94 ± 29.20	96.63 ± 41.24	<.01
Anterior segment vs inferior segment (ms)	30.31 ± 8.89	25.56 ± 11.56	.806	38.88 ± 37.03	115.63 ± 59.43	<.01
Septal segment vs lateral segment (ms)	40.44 ± 10.45	36.5 ± 18.78	.734	47.38 ± 29.89	127.31 ± 53.18	<.01

Abbreviations: EDD, end diastolic diameter; LBBP, left bundle branch area pacing; LVEF, left ventricular ejection fraction; RVP, right ventricular pacing; SPWMD, septal to posterior wall motion delay.

The 6‐segment maximum TDs to peak 2‐D longitudinal strain of the apex four‐chamber view, apex three‐chamber view, and apex two‐chamber view were longer in RVP group than those in the LBBP group (Table 2, Figure 3). The TD between the anteroseptal wall and posterior wall was statistically longer in the RVP group than in the LBBP group after pacing (22.94 ± 29.2 vs 96.63 ± 41.24 ms, *P* < .01). Comparing the TD between the anterior wall and inferior wall and the TD between septum and lateral wall, we can get similar statistical results (38.88 ± 37.03 vs 115.63 ± 59.43 ms, *P* < .01; 47.38 ± 29.89 vs 127.31 ± 53.18 ms, *P* < .01). The maximum TD was that between the septal and lateral wall in the RVP group (127.31 ± 53.18 ms). No significant TD difference of the abovementioned ventricle segments was found between the RVP group and the LBBP group at baseline. (Table 2).

Compared with RVP group, the SPWMD was significantly shorter in the LBBP group after pacing (43.31 ± 23.27 vs 93.56 ± 36.77 ms, *P* < .01) (Supplementary Figure S1). IVMD was significantly longer in the RVP group than in LBBP group (10.23 ± 3.07 vs 39.94 ± 14.81 ms, *P* < .05), and the result indicated that interventricular contractile synchronization was better in the LBBP group than in the RVP group (Supplementary Figure S2).

**FIGURE 4:**
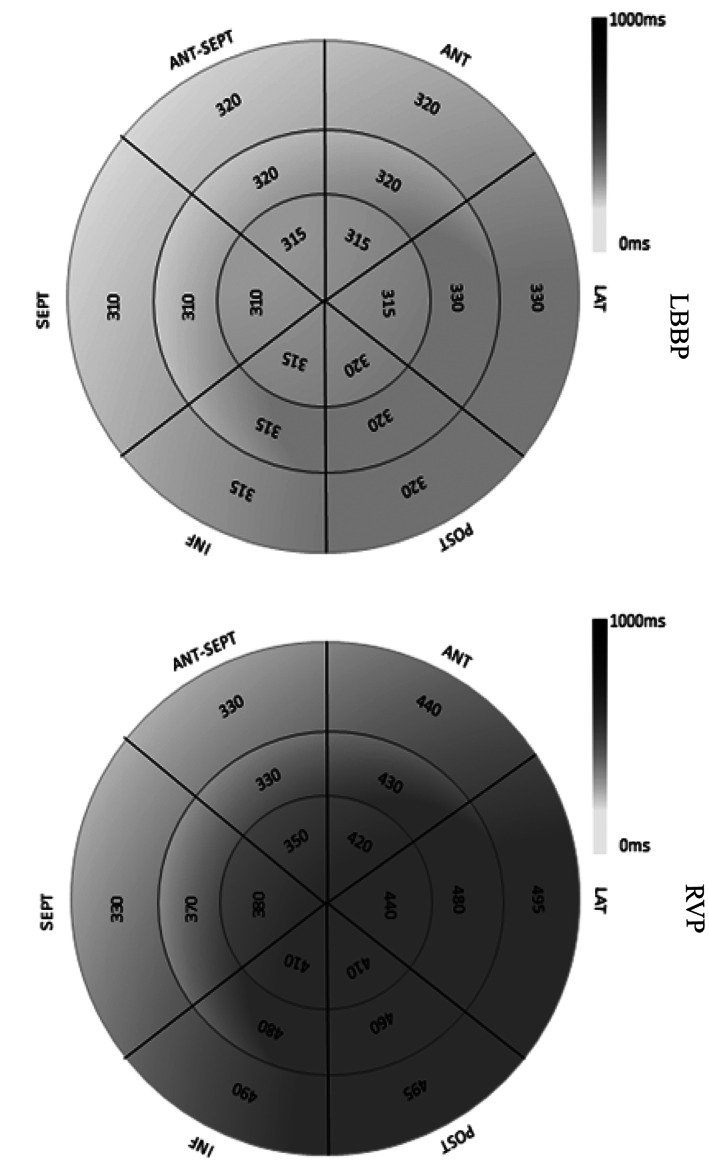
The 18‐segment time to peak left ventricular systolic strain curves exhibiting a bull's eye pattern were used to determine left ventricular synchronization status. During selective LBBP, the range of 18‐segment time to peak left ventricular systolic strain was 310 to 330 ms, and the left ventricle showed uniform gray levels, which represents good left ventricular synchronization status. During RVP, the range of 18‐segment time to peak left ventricular systolic strain was 330 ms to 495 ms, and the left ventricle showed larger gray‐scale differences, which represents worse left ventricular synchronization status compared with LBBP. 2‐D‐TD, time difference of systolic time to peak 2‐D strains; LBBP, left branch bundle pacing; PSS, peak systolic strain; RVP, right ventricular pacing

The 18‐segment times to peak left ventricular systolic strain exhibiting a bull's eye pattern were used to determine left ventricular synchronization status. The activation sequences in the LBBP group generally started from the basal septum and were conducted to the lateral wall or posterior wall. The LV activation sequence was similar in the right ventricular sepal pacing patient. During selective left branch bundle pacing, the range of 18‐segment times to peak left ventricular systolic strain was from 310 to 330 ms, and LV showed uniform gray levels, which represented good left ventricular synchronization status (Figure 4). During right ventricular sepal pacing, the range of 18‐segment times to peak left ventricular systolic strain was from 330 to 495 ms, and LV showed larger gray‐scale difference, which represented worse left ventricular synchronization status compared with LBBP (Figure 4).

### 3.4 LV function

Two‐dimensional speckle tracking was performed. Neither systolic strain at baseline (21.36 ± 2.05 vs 20.10 ± 4.26 ms, *P* > .05) nor after pacemaker implantation (20.59 ± 2.44 vs 18.90 ± 5.29 ms, *P* > .05) revealed significant differences between the RVP and LBBP groups (Table 2). No significant difference was observed in end‐diastolic diameter and LVEF% between the RVP and LBBP groups.

### 3.5 Limitations in LBBP therapy

**FIGURE 5:**
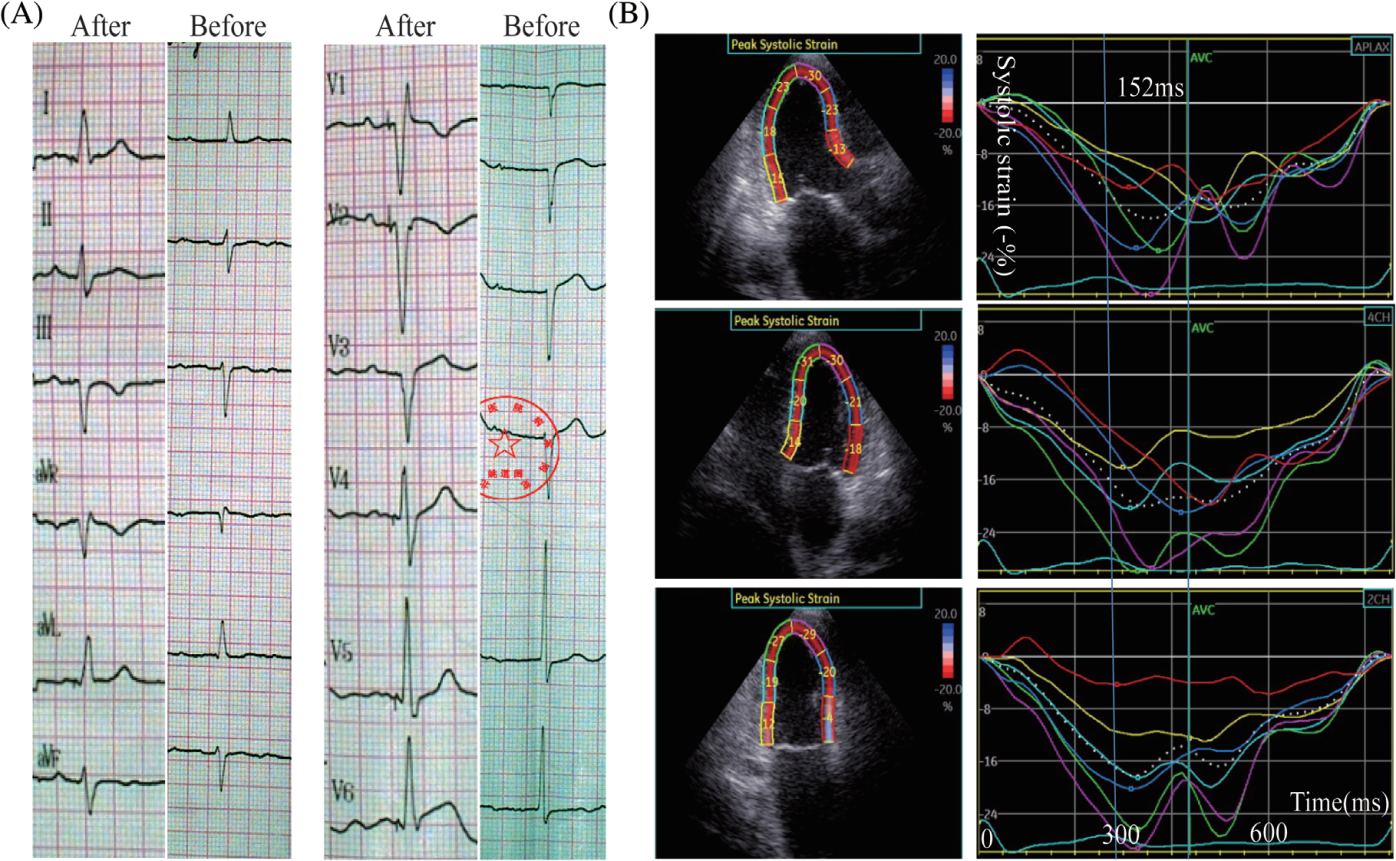
An 86 years old male patient with left anterior fascicular block and I^0^ II^0^ AVB accepted LBBP using a 3830‐pacing lead. After LBB pacing, the left anterior fascicular block still existed (Figure 5A). 2‐D speckle‐tracking echocardiography was used to get the time‐systolic strain curve of the 18 segments after operation (Figure 5B); 2D‐TDmax of the 18‐segment systolic time to peak systolic strain was 152 ms (Figure 5B), which has no statistical difference compared with that of RVP group (148.62 ± 43.67). AVB, atrioventricular block; LBBP, left bundle branch pacing; RVP, right ventricular pacing

During our study, we found that patients with intraventricular block including left anterior and posterior fascicular block cannot achieve LV contraction synchronization after pacing the left bundle branch (Figure 5).

## 4 DISCUSSION

Conventional pacing therapy places the pacemaker lead in the right ventricular apex or septum, with ventricular pacing forming a wide QRS wave and unsynchronized contraction. The His bundle‐Purkinje conduction system ensures rapid activation in both ventricles and synchronized contraction.^12^ Thus, physiological pacing should utilize the natural His bundle‐Purkinje conduction system. Compared with His bundle pacing, the threshold of LBBP distal to conduction lesion is lower and more stable; at the same time, the LBBP sensing is also significantly better than that of the His bundle. Huang W et al reported that LBBP significantly improved LVEF and LVESV in patients with heart failure and LBBB, especially in six cases with LVEF≤ 40%.^5^ Jiang et al demonstrated that patients with typical‐LBBB morphology could benefit more from LBBP for QRS correction.^13^ In our study, at LBBP the QRS wave is in the form of RBBB, but the QRS duration is significantly shorter than RVP QRS wave (108.89 ± 12.69 vs 151.00 ± 23.31 ms, *P* < .01). We fused the right and left ventricular depolarization of three patients in this article to make the LBBP QRS duration and morphology more closer to the normal QRS wave (Figure 2).

Therefore, LBBP makes the ventricular depolarization closer to the physiological state and shortens QRS duration. The ultimate goal is to obtain optimized ventricular systolic mechanical synchronization and thus maintain adequate heart function. Our study in this article aimed to evaluate ventricular systolic mechanical synchronization during permanent LBBP compared with RVP using 2D‐speckle tracking echocardiography. Both 2D‐TD_max_ and SD of the 18‐segment systolic time to PSS were significantly shorter under LBBP than under RVP, indicating that LBBP could provide better LV mechanical synchronization. As shown in the bull's eye exhibiting the 18‐segments times to peak systolic strain, the LV activation sequences during LBBP were from the septum to the lateral wall, which was consistent with the intrinsic conduction pattern. The earliest activation segments of the LV in the LBBP group were mostly in the basal and middle parts of the septum, and the latest activation segment was the lateral wall. During right ventricular sepal pacing, although the LV activation sequence was similar as the LBBP, the range of 18‐segment times to PSS was wider, and LV showed larger gray‐scale differences, which represents worse left ventricular synchronization status compared with LBBP. We also found that left and right ventricular pre‐ejection period difference which represented left and right ventricular contraction synchronization was significantly longer in the RVP group than in the LBBP group, and the result indicated that left and right ventricular contraction synchronization was better in the LBBP group than in the RVP group. However, the LVEF and average of 18‐segment PSS failed to reveal any significant differences between the LBBP group and the RVP group. This might be due to the limited observation period.

It is precisely because LBBP can bring about synchronization of ventricular contraction that more and more studies report that this technology is currently being used in the treatment of heart failure combined with left bundle branch block.^14^, ^15^ LBBP was also used in patients of atrioventricular node ablation with persistent atrial fibrillation and implantable cardioverter‐defibrillator therapy in order to improve LV function.^16^ In this study, we encountered an interesting case with atrial fibrillation, III° AVB and symptoms of heart failure including dyspnea and decreased activity tolerance. After being upgraded to LBBP using a 3830 pacing lead, the patient's symptoms of heart failure alleviated, and the NYHA heart function class improved from class III to class II about 1 month after operation (Supplementary Figure S3). For those right ventricular apex pacing patients with heart failure, upgrading ventricular lead to LBBP may improve LV systolic mechanical synchronization and alleviate heart failure.

In our study, LBBP is clinically feasible in patients, and pacing parameters were normal during pacemaker implantation and follow‐up. Former researches also got similar results.^17^

There were also limitations in LBBP therapy. Because LBBP cannot correct the intraventricular block which was distal to the left bundle branch. Thus, if the widened QRS wave and left ventricular systolic dyssynchrony was caused by intraventricular block, it cannot be corrected by LBBP.

## 5 CONCLUSIONS

LBBP was able to provide a physiologic ventricular activation pattern almost identical to the intrinsic conduction pattern, which resulted in ventricular mechanical contraction synchronization. Ventricular mechanical contraction synchronization after permanent LBBP helps to maintain good heart function and prevent detrimental impact of RVP on clinical outcomes due to ventricular mechanical dyssynchrony.

## CONFLICT OF INTEREST

We declare that there is no conflict of interest in this work.

## AUTHOR CONTRIBUTIONS

Beibing Di and Zhijun Sun contributed equally to this study.

## Supporting information


**Supplementary figure 1** SPWMD after left bundle branch pacing was shorter than right ventricle pacing indicating improved synchronization of ventricular septum and left ventricular posterior wall motion. SPWMD, septal‐to‐posterior wall motion delay.
**Supplementary Figure 2**. Left and right ventricular pre‐ejection period difference was longer in the RVP patient than LBBP patient (49 ms vs11ms), and the result indicates left and right ventricular contraction synchronization was better in the LBBP patient than the RVP patient. In the LBBP case, the left and right ventricular pre‐ejection period were 116 ms and127 ms respectively, and Left and right ventricular pre‐ejection period difference was 11 ms(=127‐116 ms). In the RVP case, the left and right ventricular pre‐ejection period were 180 ms and131 ms respectively, and Left and right ventricular pre‐ejection period difference was 49 ms(=180‐131 ms).
**Supplementary Figure 3**. The 78‐years‐old female patient with atrial fibrillation and III^0^ AVB had symptoms of heart failure including dyspnea and decreased activity tolerance. The patient underwent pacemaker implantation 10 years ago, and her ventricular pacing ratio was 100%. Because of ventricular lead malfunction (threshold>4.0v/1.0 ms) the patient was needed to implant a new ventricular lead. The former ventricular lead was in the right ventricular apex (Sup Figure 3A↑). After being upgraded to LBBP using a 3830 pacing lead (Sup Figure 3A⋆), the duration of pacing QRS wave reduced from 200 ms to 120 ms(Sup Figure 3B). 2‐D speckle‐tracking echocardiography was used to get the time‐systolic strain curve of the 18 segments; 2D‐TD_max_ of the 18‐segment systolic time to peak systolic strain was 96 ms (Sup Figure 3C), which was significantly shorter than that of RVP group (148.62 ± 43.67 ms). After LBBP, this patient's symptoms of heart failure alleviated, and the NYHA heart function class improved from class III to class II about one month after operation.

## Data Availability

The data used to support the findings of this study are available from Beibing Di (beibingyouxiang@163.com) upon reasonable request.
