# Supplementary material for: Assessment of ventricular mechanical synchronization after left bundle branch pacing using 2‐D speckle tracking echocardiography
Source: Clin Cardiol. 2020 Oct 21;43(12):1562–72. doi: 10.1002/clc.23481 (PMC7724215; doi:10.1002/clc.23481)
Supplement: Supplementary file 1 — Supplementary figure 1 SPWMD after left bundle branch pacing was shorter than right ventricle pacing indicating improved synchronization of ventricular septum and left ventricular posterior wall motion. SPWMD, septal‐to‐posterior wall motion delay. Supplementary Figure 2. Left and right ventricular pre‐ejection period difference was longer in the RVP patient than LBBP patient (49 ms vs11ms), and the result indicates left and right ventricular contraction synchronization was better in the LBBP patient than the RVP patient. In the LBBP case, the left and right ventricular pre‐ejection period were 116 ms and127 ms respectively, and Left and right ventricular pre‐ejection period difference was 11 ms(=127‐116 ms). In the RVP case, the left and right ventricular pre‐ejection period were 180 ms and131 ms respectively, and Left and right ventricular pre‐ejection period difference was 49 ms(=180‐131 ms). Supplementary Figure 3. The 78‐years‐old female patient with atrial fibrillation and III0 AVB had symptoms of heart failure including dyspnea and decreased activity tolerance. The patient underwent pacemaker implantation 10 years ago, and her ventricular pacing ratio was 100%. Because of ventricular lead malfunction (threshold>4.0v/1.0 ms) the patient was needed to implant a new ventricular lead. The former ventricular lead was in the right ventricular apex (Sup Figure 3A↑). After being upgraded to LBBP using a 3830 pacing lead (Sup Figure 3A⋆), the duration of pacing QRS wave reduced from 200 ms to 120 ms(Sup Figure 3B). 2‐D speckle‐tracking echocardiography was used to get the time‐systolic strain curve of the 18 segments; 2D‐TDmax of the 18‐segment systolic time to peak systolic strain was 96 ms (Sup Figure 3C), which was significantly shorter than that of RVP group (148.62 ± 43.67 ms). After LBBP, this patient's symptoms of heart failure alleviated, and the NYHA heart function class improved from class III to class II about one month after operation. [file CLC-43-1562-s001.docx]

Supplementary data


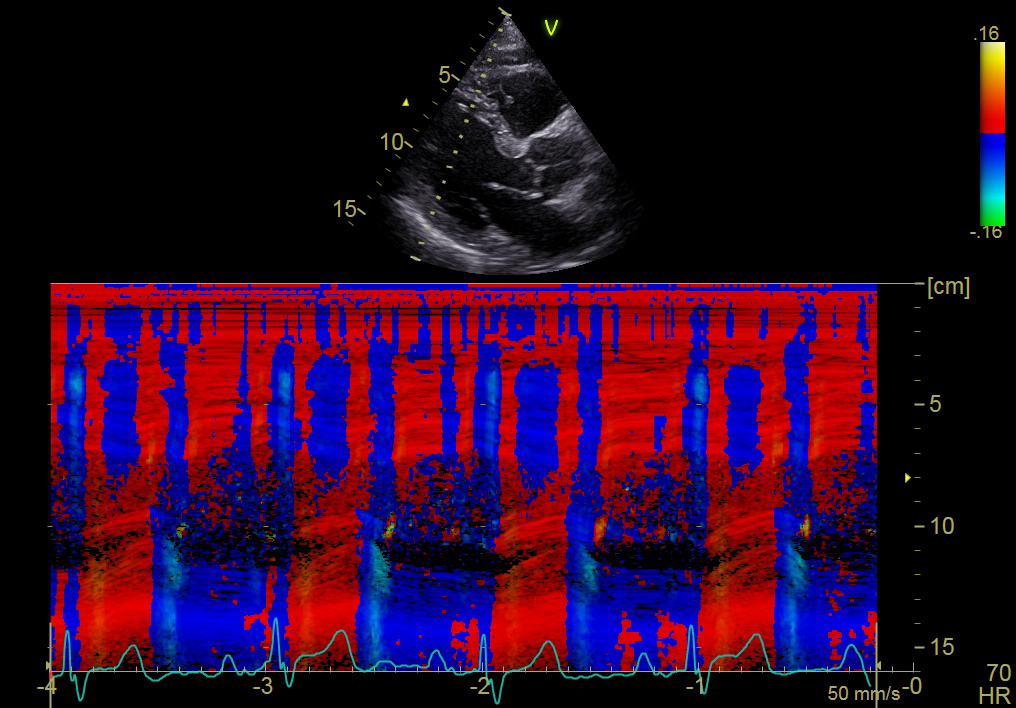


**40ms**


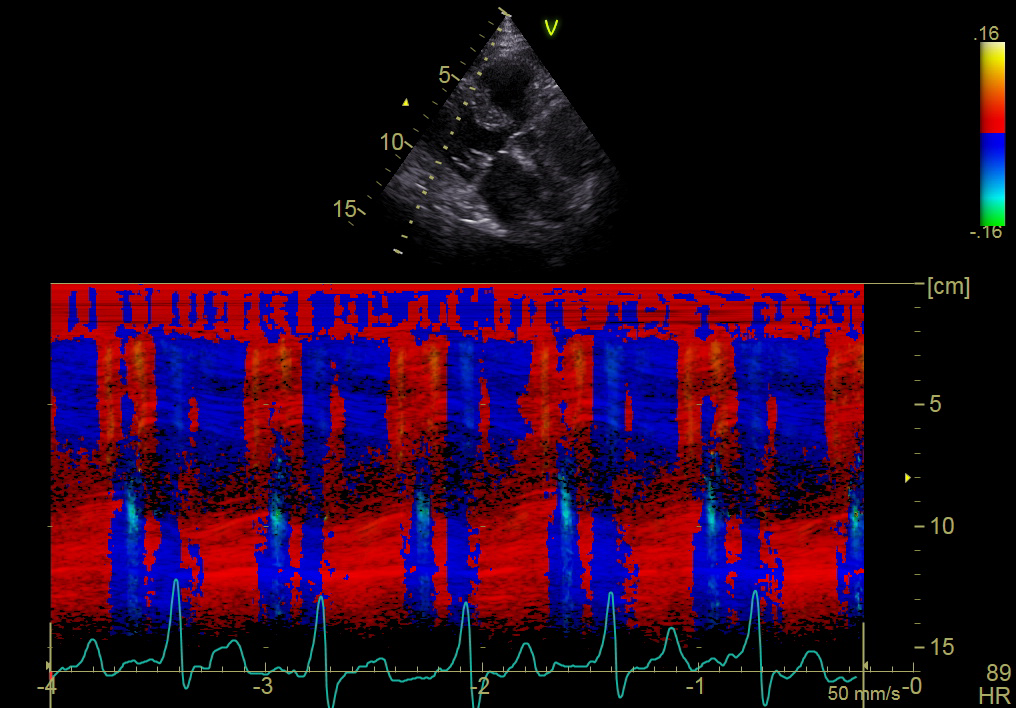


**130ms**

Supplementary figure 1. SPWMD after left bundle branch pacing was shorter than right ventricle pacing indicating improved synchronization of ventricular septum and left ventricular posterior wall motion. SPWMD, septal-to-posterior wall motion delay.


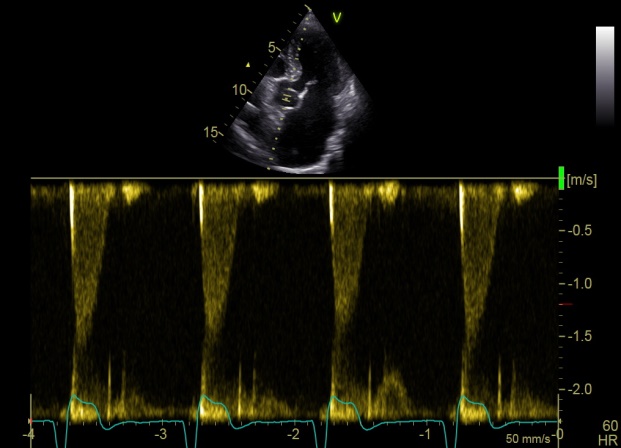

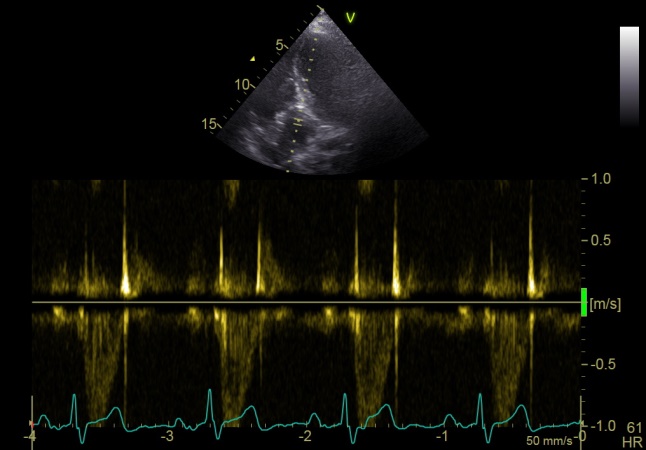


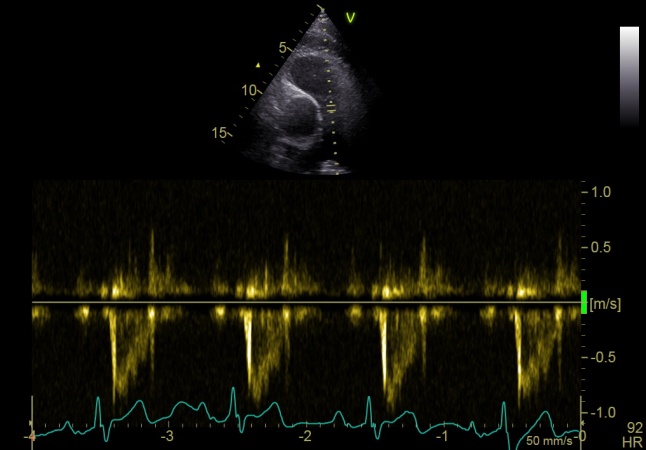

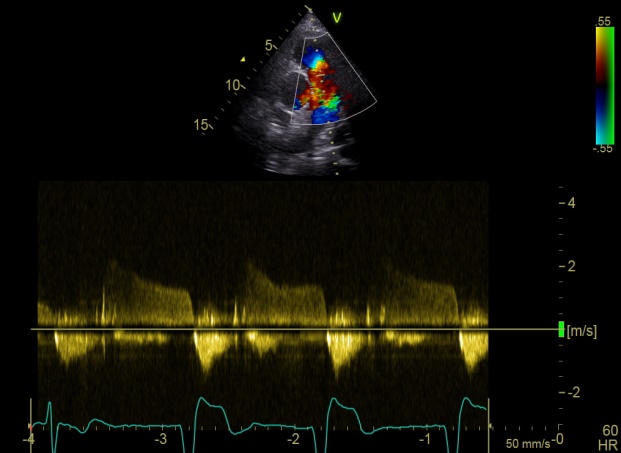


**RVP**

**LBBP**

**Left ventricular pre-ejection period**

180ms

116ms

**Right ventricular pre-ejection period**

131ms

127ms

Supplementary figure 2. Left and right ventricular pre-ejection period difference was longer in the RVP patient than LBBP patient (49ms vs11ms), and the result indicates left and right ventricular contraction synchronization was better in the LBBP patient than the RVP patient. In the LBBP case, the left and right ventricular pre-ejection period were 116ms and127 ms respectively, and Left and right ventricular pre-ejection period difference was 11ms(=127-116ms). In the RVP case, the left and right ventricular pre-ejection period were 180ms and131 ms respectively, and Left and right ventricular pre-ejection period difference was 49ms(=180-131ms).

127ms


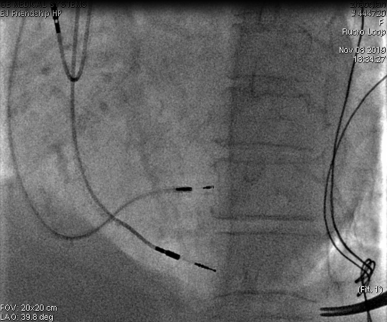


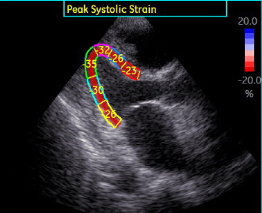

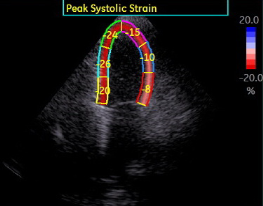

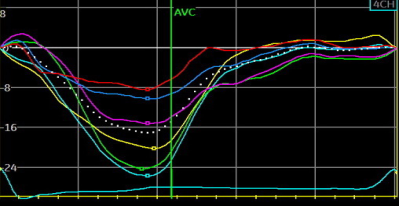

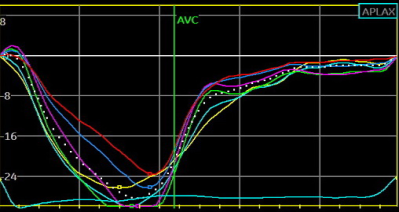

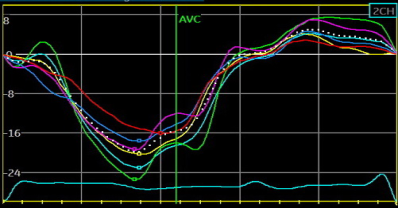

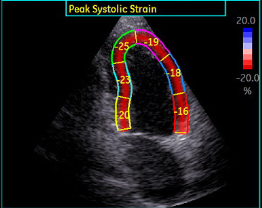


96ms

Systolic strain (-%)

**A**


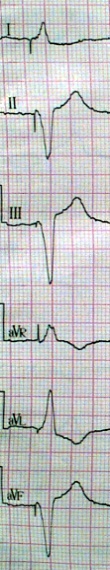

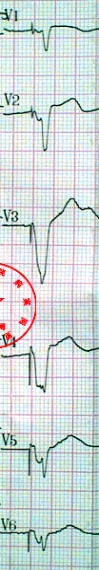

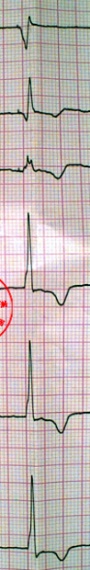

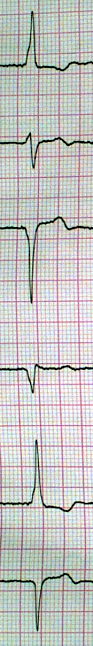


RVP LBBP RVP LBBP

Time (ms)

800

400

0

**C**

**B**

Supplementary figure 3. The 78-years-old female patient with atrial fibrillation and III⁰ AVB had symptoms of heart failure including dyspnea and decreased activity tolerance. The patient underwent pacemaker implantation 10 years ago, and her ventricular pacing ratio was 100%. Because of ventricular lead malfunction (threshold>4.0v/1.0ms) the patient was needed to implant a new ventricular lead. The former ventricular lead was in the right ventricular apex (Sup Fig 3A↑). After being upgraded to LBBP using a 3830 pacing lead (Sup Fig 3A☆), the duration of pacing QRS wave reduced from 200ms to 120ms(Sup Fig 3B). 2-D speckle-tracking echocardiography was used to get the time-systolic strain curve of the 18 segments; 2D-TD_max_ of the 18-segment systolic time to peak systolic strain was 96ms (Sup Fig 3C), which was significantly shorter than that of RVP group (148.62±43.67 ms). After LBBP, this patient’s symptoms of heart failure alleviated, and the NYHA heart function class improved from class III to class II about one month after operation.
